# Baicalin Liposome Alleviates Lipopolysaccharide-Induced Acute Lung Injury in Mice via Inhibiting TLR4/JNK/ERK/NF-*κ*B Pathway

**DOI:** 10.1155/2020/8414062

**Published:** 2020-11-11

**Authors:** Yu Long, Yan Xiang, Songyu Liu, Yulu Zhang, Jinyan Wan, Qiyue Yang, Mingquan Cui, Zhimin Ci, Nan Li, Wei Peng

**Affiliations:** ^1^School of Pharmacy, Chengdu University of Traditional Chinese Medicine, Chengdu 611137, China; ^2^Hospital of Chengdu University of Traditional Chinese Medicine, Chengdu, China

## Abstract

Acute lung injury (ALI) and acute respiratory distress syndrome (ARDS) are challenging diseases with the high mortality in a clinical setting. Baicalin (BA) is the main effective constituent isolated from the Chinese medical herb *Scutellaria baicalensis* Georgi, and studies have proved that it has a protective effect on ALI induced by lipopolysaccharide (LPS) due to the anti-inflammatory efficacy. However, BA has low solubility which may limit its clinical application. Hence, we prepared a novel drug delivery system—Baicalin liposome (BA-LP) in previous research—which can improve some physical properties of BA. Therefore, we aimed to explore the effect of BA-LP on ALI mice induced by LPS. In pharmacokinetics study, the values of *t*_1/2_ and AUC_0-__*t*_ in the BA-LP group were significantly higher than that of the BA group in normal mice, indicating that BA-LP could prolong the duration time *in vivo* of BA. The BA-LP group also showed a higher concentration in lung tissues than the BA group. Pharmacodynamics studies showed that BA-LP had a better effect than the BA group at the same dosage on reducing the W/D ratio, alleviating the lung injury score, and decreasing the proinflammatory factors (TNF-*α*, IL-1*β*) and total proteins in bronchoalveolar lavage fluids (BALF). In addition, the therapeutic effects of BA-LP showed a dose-dependent manner. Western blot analysis indicated that the anti-inflammatory action of BA could be attributed to the inhibition of the TLR4-NF*κ*Bp65 and JNK-ERK signaling pathways. These results suggest that BA-LP could be a valuable therapeutic candidate in the treatment of ALI.

## 1. Introduction

Acute lung injury (ALI) is one of the clinically common severe respiratory infection diseases caused by various intrapulmonary and extrapulmonary pathogenic factors [1]. The more severe diffuse lung injury is called acute respiratory distress syndrome (ARDS). ARDS can cause multiple organ dysfunction syndromes in the later stage, which has an extremely serious damage to the human body. ALI, as a basic pathophysiological change of ARDS, is a challenging disease in the clinical setting [2]. It is characterized by the damage of the alveolar capillary system, increased pulmonary vascular permeability, diffuse alveolar injury, intra-alveolar fibrin, interstitial edema, and inflammatory cell infiltration [3]. Although ALI/ARDS has been studied in recent years, there is still a lack of effective ways to reduce its high mortality. Hence, it is urgent to develop a novel effective therapy to intervene in the diseases.

Lipopolysaccharide (LPS) is a pathogenic endotoxin of the outer membranes of Gram-negative bacteria, which is widely used in preparing animal models of ALI [4]. LPS can induce a series of inflammatory responses by the release of some inflammatory cytokines and factors [5]. The emergence and disappearance of inflammation are unique functions of the immune system, which represents the natural process of the body to repair the injury and remove pathogenic substances. Some inflammatory responses controlled are conducive for the clearing of harmful substances, while some without control may cause fatal injury to the body, such as the inflammation during ALI, which plays an extraordinary role in the course of disease [6, 7]. Hence, it is very important to take measures to eliminate inflammation for the treatment of ALI.

Baicalin (5,6-dihydroxy-7-O-glucuronide flavone, BA) is the main effective constituent isolated from the roots of the Chinese medical herb *Scutellaria baicalensis* Georgi, which is widely used in pneumonia, hepatitis, atherosclerosis [8], and other conditions. Recent studies have indicated that BA has many pharmacological activities, like anti-inflammation [9], antioxidative stress [10], neuroprotective [11], and antibacterial [12]. Increasing researches have showed that BA has a protective effect on ALI induced by LPS [13]. However, BA has a low solubility due to the existence of glycosyl groups in the molecular structure. And its bioavailability is only 2.2% after oral administration [14], which limits the development and the clinical use of it. Therefore, it is important for us to find a new way to improve some properties of BA.

In the previous study, we prepared a novel drug delivery system, Baicalin liposome (BA-LP) [15], to improve some properties of BA. The data showed that the cumulative release rate of BA-LP *in vitro* was about 57.43%, while the BA was 95.4%. Therefore, we aimed to explore the effect of BA-LP against the LPS-induced ALI. So, in this research, we researched the pharmacokinetics of BA-LP in normal mice. Then, we compared the therapeutic effects of BA and BA-LP on LPS-induced ALI and explored the underlying molecular mechanisms associated with inflammation by the regulation of the TLR4/MARKs/NF-*κ*B signaling pathways. In this study, we hope to offer some references for the treatment of ALI in the future.

## 2. Materials and Methods

### 2.1. Reagents

BA (purity over 90%) was purchased from Nanjing Zelang Biotechnology Co., Ltd. (Nanjing, China). The BA standard agent (No. MUST-19010408, purity over 98%) was obtained from Chengdu Man Site Biotechnology Co., Ltd. (Chengdu, China). The Rutin (RU) standard agent (No. L-001-181216) was purchased from Chengdu Herbpurify Co., Ltd. (Chengdu, China). Cholesterol (No. B80859) and soybean lecithin (No. SY-S1-190601) purchased from A.V.T (shanghai, China) Pharmaceutical Co., Ltd. (Shanghai, China). Lipopolysaccharide (LPS) L2880 was obtained from Sigma-Aldrich Co., Ltd. (Missouri, USA). BCA protein analysis kit (No. P0012S) was obtained from Beyotime Biotechnology Co., Ltd. (Shanghai, China). Mouse TNF-*α* ELISA kit (96T, NO. 21I333) and Mouse IL-1*β* ELISA kit (96T, NO21T361) were purchased from Excell Bio Co., Ltd. (Shanghai, China). Primary antibodies against TLR4, p-JNK, and p-ERK were purchased from Affinity Biosciences, OH, USA. p-p65 (phospho S536), p65, ERK, and *β*-actin were all from Abcam (Cambridge, Britain). JNK was obtained from Proteintech Group, Inc. (Chicago, USA). HRP-Goat anti Rabbit and HRP-Goat anti mouse were both from Abcam (Cambridge, Britain). Water was ultrapure water, methanol and acetonitrile were chromatographic grade, and other reagents were analytical grade.

### 2.2. Animals

Male KM mice, weighing between 20 and 25 g, were provided by Chengdu Dashuo Laboratory Animal Co., Ltd. (Chengdu, China). The mice were housed in a stabilized laboratory condition with free food and water, the temperature at approximately 24 ± 1°C, and 40% to 80% relative humidity. All procedures in this experiment were performed in accordance with the Code of Ethics of the World Medical Association (Declaration of Helsinki). And this study protocol was approved by the Chengdu University of Traditional Chinese Medicine Animal Ethical Experimentation Committee with the approval no. 2016KL-027.

### 2.3. Preparation of BA-LP

BA-LP was prepared by reverse evaporation as our previous research [15]. In short, cholesterol (35 mg) and soybean lecithin (200 mg) were fully dissolved in a mixture of chloroform and diethyl ether (1 : 2) to form an organic phase. Then, BA phosphate buffer solution (PBS) (5.2 mg/mL) was injected into the organic at a uniform speed. Put the mixture into the ultrasonic homogenizer (10 min, 20°C) to form an emulsion liquid. After the organic solvent was removed by rotating evaporation, 1 mL PBS was added to hydrate until a yellow and clear solution was formed; then, the BA-LP was obtained.

### 2.4. Pharmacokinetics and Tissue Distributions of BA-LP and BA in Normal Mice

#### 2.4.1. Preparation of Plasma and Tissue Samples

Mice were randomly divided into the free-BA and BA-LP groups (100 mg/kg BA; *n* = 5 mice at each time point). The two groups were intravenously administered with drugs via the tail vein, separately. Blood samples were collected from the submandibular vein with heparinized microblood collecting tubes at 5, 15, 30, 60, 90, 120, 240, 360, and 480 min after administration and centrifuged at 3500 rpm for 10 min. Plasma was collected and stored at -20°C until further analysis. After euthanasia, their livers, hearts, spleens, lungs, and kidneys were quickly harvested, the blood in the surface was washed with normal saline, and then, they were dried with filter paper, which were collected for BA concentration measurement.

After weighing, the tissues were homogenized with normal saline as the ratio 1 : 2 (weight: volume), except the spleens as 1 : 4 (weight: volume). All the plasma and tissues homogenate samples were treated as follows: 300 *μ*L samples were placed into centrifuge tubes and vortexed for 1 min with 60 *μ*L hydrochloric acid (1 mol/L). After adding 40 *μ*L internal standard (RU) and 1.2 mL acetonitrile, the mixture was vortexed for 3 min and then centrifuged at 12000 rpm for 10 min. The obtained supernatant was evaporated under nitrogen atmosphere in a water bath at 36°C. The residue was dissolved in 100 *μ*L methanol (70%) and vortexed for 5 min and centrifuged at 15000 rpm for 10 min. Then, we get the supernatant, and 20 *μ*L solution was injected into the HPLC system.

All the samples were measured on a high-performance liquid chromatography (HPLC) system (Thermo Fisher Scientific, USA) via an AQ-C_18_ (4.6 × 250mm) analytical column. The HPLC conditions were methanol-water-phosphoric acid (41 : 59 : 0.2, *v*/*v*/*v*) at the flow rate of 1 mL/min, detection wavelength for 276 nm, and a column temperature of 35°C.

#### 2.4.2. Validation of HPLC Analytical Method

*(1) Preparation of Related Solutions*. The BA standard was precisely weighed and dissolved with methanol to obtain a stock solution of 1516 *μ*g/mL and diluted to 485.12, 194.048, 97.024, 48.512, 19.4048, 9.7024, 4.8512, 2.4256, and 1.4554 *μ*g/mL with methanol.

The RU reference was accurately weighed to prepare a stock solution with a concentration of 501.6 *μ*g/mL with methanol. And the solution was diluted with methanol to prepare the stock solution of 100.32 *μ*g/mL, which was the internal standard working solution.

*(2) Specificity and Linearity*. Full validation of the method was performed in blank plasma and tissue homogenate of mouse. Taking them with the BA reference substance and RU reference substance to prepare samples, the specificity of the method was evaluated based on whether the plasma and tissue endogenous substances had influence on the BA and RU.

Taking the blank plasma (300 *μ*L) or tissue homogenate (300 *μ*L) with the BA working solution (80 *μ*L) of different concentrations, please refer to Section 2.4.1 for the next steps. The prepared sample was injected into HPLC for recording the peak area of BA and RU, and then, the ratio of BA to RU peak area (*y*) was used to regress the mass concentration of BA (*x*) with the least-square method to obtain the standard curve.

*(3) Precision and Accuracy*. Three BA concentrations were selected as the high, medium, and low concentrations. After preparation, the sample was injected 5 times within one day to obtain intraday precision. The interday precision was obtained for 3 days. The precision was evaluated as the relative standard deviation (RSD), and accuracy was expressed as the relative error (RE).

### 2.5. Modeling and Grouping

All mice were randomly divided into the following six groups: the control group, LPS group, LPS+BA (100 mg/kg) group, and LPS+BA-LP (50, 100, and 200 mg/kg) group. Different drug intervention groups were given intravenous injection of drugs daily for 3 days. One hour after the last administration, all animals, except the control group, received intratracheal drip of LPS (8 mg/kg) to induce the ALI models. The control group was intranasally received same normal saline. After 12 h of LPS stimulation, the mice were euthanized via cervical dislocation with chloral hydrate solution anesthetized. The bronchoalveolar lavage fluid (BALF) and lung tissues were obtained and stored for further experiments.

### 2.6. Lung Wet/Dry (W/D) Weight Ratio

The fresh lungs were collected and weighed to obtain wet weight. Then, the lungs were dehydrated at 80°C for 48 h [16] until weight remained unchanged to get the dry weight. Then, the W/D ratio of lung was calculated.

### 2.7. Histopathologic Evaluation of Lung Tissues

The lung tissues were obtained and fixed with 10% neutral formaldehyde. Lung tissues were treated by dehydration, pruning, embedding, sectioning, hematoxylin and eosin staining, sealing, and finally microscopic examination [17]. The severity of microscopic injury was graded from 0 (normal) to 5 (severe) in the following categories: interstitial edema, hemorrhage, hyaline membrane formation, necrosis, and congestion.

### 2.8. Determination of Protein and Inflammatory Factors in the BALF

After being sacrificed, the muscles in the neck of mice were separated to expose the trachea. And a catheter was inserted into the trachea via tracheostomy. The lung was lavaged with PBS, and then, the lavage liquid was obtained. The concentration of protein in BALF can be used as an indicator of the increase of alveolar capillary barrier permeability, which can be measured by BCA protein analysis kit. The determination of TNF-*α* and IL-1*β* levels were detected by ELISA.

### 2.9. Western Blot Analysis

After the mice were euthanized, the whole lungs were collected, We grind the obtained tissue and extract the protein. The protein contents in the supernatant of the cracked lung were detected by BCA protein analysis kit. Then, samples were isolated from 10% SDS-polyacrylamide gel and transferred to the polyvinylidene difluoride (PVDF) membrane for protein detection. Blocking the membranes in 5% nonfat milk dissolved in TBST for 1 h at 25°C, and then, membranes were incubated with an antibody. After dropping the fresh ECL solution to the membrane with protein side, the protein bands were detected by AlphaEaseFC software.

### 2.10. Statistical Analyses

All values are expressed as the means ± SEM. DAS3.0 (Mathematical Pharmacology Professional Committee of China, Shanghai, China) was used to calculate the in vivo pharmacokinetic parameters by a noncompartmental model. The statistical package was performed on SPSS 22.0 (SPSS Inc., Chicago, IL, USA) software. Differences between groups were analyzed using a one-way ANOVA (Dunnett's *t*-test) and two-tailed Student's *t*-test. The results were considered statistically significant at *P* < 0.05.

## 3. Results

### 3.1. Pharmacokinetics Study

#### 3.1.1. Method Validation

As shown in Table 1, the calibration curve of BA in the mouse plasma and tissue homogenate was determined in a certain range, and the correlation coefficient was greater than 0.990, showing the good linearity.

As Table 2 shows, both RSD% in the intraday and interday precisions of these concentrations of the plasma and tissues were less than 15%, indicating that the method had a good precision. And the intraday accuracy range was 95.32-101.65%, while the interday accuracy range of BA in three concentrations were 98.19-101.29%, which are in accordance with the guide.

#### 3.1.2. Pharmacokinetics of Baicalin in Plasma of Mice

**Figure 1:**
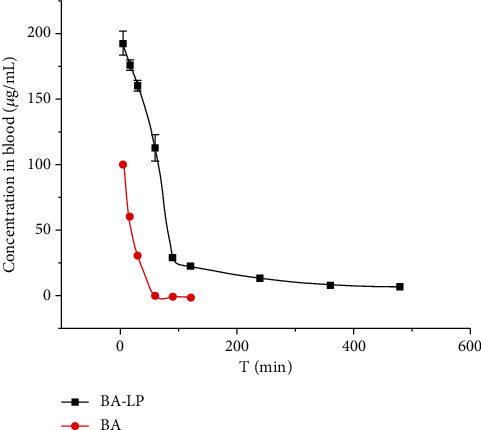
The plasma concentration-time profile of BA after *i.v.* administration of BA and BA-LP (100 mg/kg) in normal mice (*n* = 5).

The plasma concentration of BA and BA-LP was investigated by using normal mice.  Figure 1 showed that the plasma BA concentration versus time profiles in mouse plasma following *i.v.* administration of BA-LP and BA in normal mice at a dose of 100 mg/kg. As shown in Table 3, the AUC_0-__*t*_ of BA-LP was about 6.46 times of BA (17536.084 ± 928.392 mg/L^∗^min vs. 2712.911 ± 55.945 mg/L^∗^min), which was significantly higher than that of BA (*P* < 0.05), indicating that BA-LP had a better absorption than BA in mice. The MRT_0-__*t*_ of BA-LP was higher than BA (*P* < 0.001), and the *t*_1/2_ values of the BA-LP and BA groups were 202.963 ± 18.291 min and 127.18 ± 10.849 min, respectively, which meant that the retention time of BA-LP was longer than that of BA. The *V*_d_ can reflect the distribution of BA in the tissue, and *V*_d_ value of BA-LP was about 1/4 of BA (1.500 ± 0.168 L/kg vs. 6.209 ± 0.582 L/kg, *P* < 0.05), indicating that BA-LP was easier to enter the blood circulation than tissue.

#### 3.1.3. Tissue Distributions

As shown in Table 4, the distributions of BA were detected in the heart, liver, spleen, lung, and kidney of normal mice after intravenous injection of BA-LP and BA at 100 mg/kg. These results indicated that the distributions of BA in the heart, liver, spleen, and lung increased appreciably, while the content of BA in the kidney reduced relatively after the injection of BA-LP compared with BA. The BA targeting parameters of Te, TI, Ce, and RTE in different tissues after intravenous administration with BA-LP and BA in normal mice were shown in Table 5. We can find that BA-LP had certain targeting to the liver, heart, spleen, and lung, and the highest is to the liver, from these targeting parameters. Te indicated the selective targeting of the drug delivery system to a tissue; the larger the Te, the more targeted the drug delivery system is to the tissue. If TI > 1, it meant that the drug had targeting effects on some tissues; the higher the TI value, the greater the targeting. RTE can reflect the tendency distributions of different preparations of the same drug to tissues. The positive value of RTE indicates that it has a strong targeting effect on the tissue; otherwise, it has no targeting effect.

### 3.2. The Effect of Baicalin on Lung W/D Ratio

**Figure 2:**
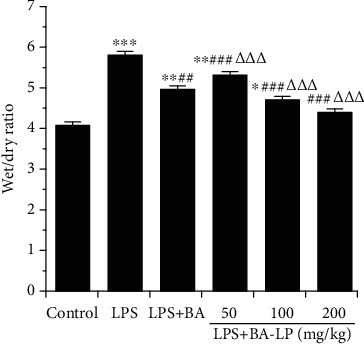
BA alleviates the lung W/D ratio induced by LPS. After 12 h after LPS injection, mice were sacrificed to get the lung tissues to analyze the lung wet/dry ratio. The values were presented as means ± SD (*n* = 5). ^∗^*P* < 0.05, ^∗∗^*P* < 0.01, and ^∗∗∗^*P* < 0.001 vs. the control group; ^##^*P* < 0.01 and ^###^*P* < 0.001 vs. the LPS group; and ^△△^*P* < 0.01 and ^△△△^*P* < 0.001 vs. the LPS+BA group.

In this study, the pulmonary edema was evaluated by measuring the lung W/D ratio (Figure 2). The results showed that the lung W/D ratio was significantly higher than the control group after LPS administration (*P* < 0.05). However, after intervention of the BA, the W/D ratio was significantly reduced (*P* < 0.05), which showed that the BA could protect the lung injury. While at the same dose (100 mg/kg), BA-LP was more effective than BA in reducing the W/D ratio (*P* < 0.001). Furthermore, the high dosage of BA showed a much better therapeutic effect than the low dosage group (*P* < 0.01), indicating that the protective effect was dose dependent.

### 3.3. The Effect of Baicalin on Histopathology Changes of Lung Tissues

**Figure 3:**
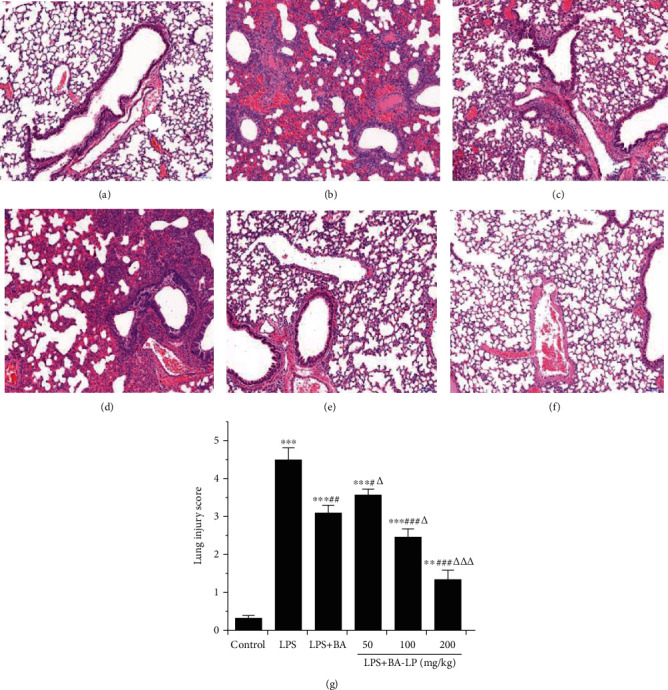
The effect of BA on lung tissue histopathological changes. Histopathologic sections of lung tissues (H&E, ×100): (a) control group, (b) LPS group, (c) LPS+BA (100 mg/kg), (d) LPS+BA-LP (50 mg/kg), (e) LPS+BA-LP (100 mg/kg), and (f) LPS+BA-LP (200 mg/kg) mice. (g) The pathological scores was evaluated during ALI. The values were presented as means ± SD (*n* = 5). ^∗^*P* < 0.05, ^∗∗^*P* < 0.01, and ^∗∗∗^*P* < 0.001 vs. the control group; ^#^*P* < 0.05, ^##^*P* < 0.05, and ^###^*P* < 0.01 vs. the LPS group; and ^△^*P* < 0.05, ^△△^*P* < 0.01, and ^△△△^*P* < 0.001 vs. the LPS+BA group.

To evaluate the pathological changes of the lung tissues, hematoxylin and eosin (H&E) staining and a lung injury score system were carried out in this study. As shown in Figure 3, the lung tissues of the LPS group showed some obvious inflammation alterations, such as inflammatory cell infiltration, alveolar wall thickening, pulmonary congestion, and fibrous hyperplasia, while there were no obvious pathological changes in the control group. After treatment, BA markedly alleviated the lung injury score induced by LPS (*P* < 0.05) in a dose-dependent manner. In addition, BA-LP showed a better effect than BA in reducing the pathological changes at the same dose (100 mg/kg). The scores was shown in Figure 3(g), the mean pathological score was significantly increased after LPS-induced ALI compared with the control group (*P* < 0.05), while BA intervention groups could reduce the pathological score.

### 3.4. The Effect of BA on Protein Concentrations in BALF

**Figure 4:**
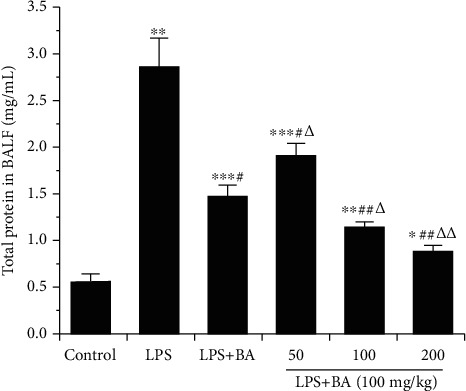
BA alleviates the total proteins in BALF induced by LPS. After 12 h of LPS administration, mice were sacrificed to get the BALF. The values were presented as means ± SD (*n* = 4). ^∗^*P* < 0.05, ^∗∗^*P* < 0.01, and ^∗∗∗^*P* < 0.001 vs. the control group; ^#^*P* < 0.05 and ^##^*P* < 0.01 vs. the LPS group; and ^△^*P* < 0.05 and ^△△^*P* < 0.01 vs. the LPS+BA group.

To determine the levels of proteins and cytokines in BALF, BALF was collected 12 h after LPS challenge. As shown in Figure 4, the total protein concentrations in BALF were significantly increased after LPS administration (*P* < 0.01). BA efficiently reduced the protein concentration in varied doses (50, 100, and 200 mg/kg), and BA-LP had a better therapeutic effect than BA at the same dosage of BA (100 mg/kg, *P* < 0.05).

### 3.5. The Effects of BA on TNF-*α* and IL-1*β* in BALF

**Figure 5:**
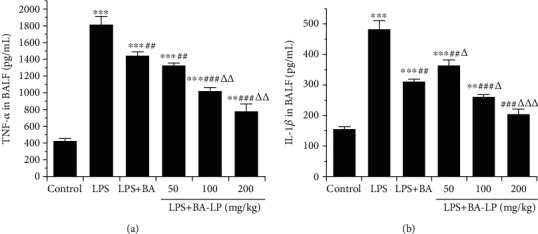
Effect of BA on the levels of TNF-*α* (a) and IL-1*β* (b) in BALF. The values were presented as means ± SD (*n* = 5). ^∗∗^*P* < 0.01 and ^∗∗∗^*P* < 0.001 vs. the control group; ^##^*P* < 0.01 and ^###^*P* < 0.001, vs. the LPS group; and ^△^*P* < 0.05, ^△△^*P* < 0.01, and ^△△△^*P* < 0.001 vs. the LPS+BA group.

The effect of BA on TNF-*α* and IL-1*β* production in BALF were analyzed at 12 h after LPS challenge by ELISA kits. As shown in Figure 5, the concentrations of TNF-*α* and IL-1*β* in BALF were significantly increased after LPS injection (*P* < 0.01), while BA can significantly reduce the content of inflammatory factors TNF-*α* (*P* < 0.05) and IL-6 (*P* < 0.05). In addition, the inhibitory effects of BA-LP on TNF-*α* and IL-1*β* were much better than that of BA at 100 mg/kg (*P* < 0.01, *P* < 0.05). In addition, the LPS+BA (100 mg/kg) group had no significant difference with the LPS+BA-LP (50 mg/kg) group.

### 3.6. The Effect of BA on TLR4/JNK/ERK/NF-*κ*B Signaling Pathway

**Figure 6:**
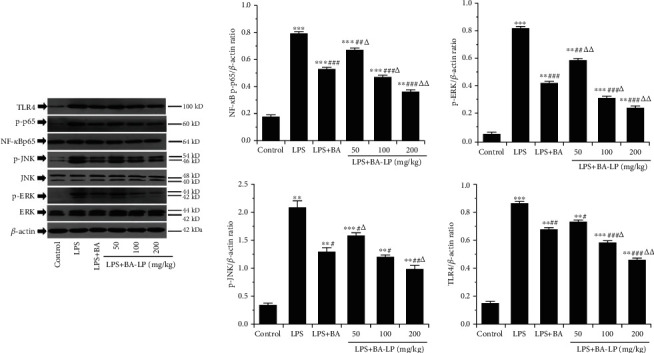
Effects of BA on the TLR4/JNK/ERK/NF-*κ*B signaling pathway. The expressions of TLR4 protein; phosphorylation of NF-*κ*Bp65, JNK, and ERK; and total NF-*κ*Bp65, JNK, and ERK in lung tissues of mice were determined by western blotting. *β*-Actin was used as an internal control. The values were presented as means ± SD (*n* = 3). ^∗^*P* < 0.05, ^∗∗^*P* < 0.01, and ^∗∗∗^*P* < 0.001 vs. the control group; ^#^*P* < 0.05, ^##^*P* < 0.01, and ^###^*P* < 0.001 vs. the LPS group; and ^△^*P* < 0.05 and ^△△^*P* < 0.01 vs. the LPS+BA group.

TLR can induce inflammatory responses after being activated by LPS, and NF-*κ*B is located at the key position of the TLR downstream signaling pathway. The TLR4-NF-*κ*B signaling pathway plays an important role in the regulation of LPS-induced ALI [18–21]. MAPK signaling pathways are also important ways of TLR4 initiation [22], which are regulated by a characteristic phosphorelay system in which a series of protein kinases phosphorylate, such as JNK and ERK [23]. We detected some key proteins using western blot experiments to explore the mechanisms of BA treatment on ALI. After 12 h of LPS injection, the expression of TLR4 and the phosphorylation of NF-*κ*Bp65, JNK, and ERK were markedly increased (Figure 6, *P* < 0.01, *P* < 0.01, *P* < 0.01, and *P* < 0.01). Additionally, there was no difference in the expression of total NF-*κ*Bp65, JNK, and ERK among these groups. As shown in Figure 6, treatment with BA could inhibit the phosphorylation of NF-*κ*Bp65, JNK, and ERK. While there was no significant difference between the LPS+BA (100 mg/kg) and LPS+BA-LP (50 mg/kg) groups in the TLR4/*β*-actin ratio, the same as the LPS+BA (100 mg/kg) and LPS+BA-LP (100 mg/kg) groups in the p-JNK/*β*-actin ratio, which indicated that BA may regulate inflammatory response via suppressing the TLR4/JNK/ERK/NF-*κ*B signaling pathway.

## 4. Discussion

BA is widely used in the treatment of infection, pneumonia, hepatitis, and other diseases. But it has a low solubility and is easy to be eliminated *in vivo*. When BA-LP was injected into mice via the vein, the predominant targeting organ is the liver, and the drug distribution in the kidney is less, which greatly decreases the nephrotoxicity. It is reported that BA has a certain intervention effect on ALI due to its notable anti-inflammatory effects. However, BA has a low bioavailability and short half-life *in vivo*, which are easy to eliminate. Passive targeting can be achieved by controlling the particle size of liposomes. Liposomes with a particle size greater than 5 *μ*m are easy to be trapped by the capillaries of the lungs. Encapsulating drugs in large-particle liposomes can achieve their accumulation. Therefore, it is helpful that making BA into liposomes to increase the targeting of drugs in lung lesions. Pharmacokinetic parameters showed that BA-LP had a longer half-life and more retention dosage than BA. In addition, the concentration of BA-LP in the lung tissue increased compared with that of BA, which suggested that BA-LP may be more efficient than BA in the treatment of lung diseases.

LPS is located on the surface of Gram-negative bacilli. The compound formed by LPS and its receptor can activate the CD14 [24] receptor on monocyte, macrophage, and other cells and lead to the production of inflammatory mediators. Therefore, LPS was commonly used to establish the animal models of ALI. Inflammation plays an important role in the course of ALI [25, 26], so we treat the ALI from the perspective of an anti-inflammatory effect.

The basic pathophysiological change of ALI/ARDS is noncardiogenic pulmonary edema caused by the increase of pulmonary capillary endothelial permeability and alveolar epithelium [27]. The edema fluid is rich in protein, and many kinds of inflammatory cells mainly composed of neutrophils. Inflammatory cells, alveolar epithelial cells and fibroblasts can produce a variety of cytokines, which can aggravate the inflammatory process and cause lung injury [4]. In this study, we observed that the W/D of the lung tissue of LPS-induced mice significantly increased, while the pulmonary edema of model animals was significantly improved under the intervention of BA. Total protein and inflammatory factors of the ALI mice in BALF were all increased, which indicated that the inflammation was produced after the established model, while the inflammation was significantly alleviated by the treatment of BA. The H&E-stained histopathological tissue section can show the degree of lung injury. After BA intervention, the degree of lung injury in ALI mice was significantly reduced. In the whole experiment, it was found that the therapeutic effect of BA-LP was better than that of BA at the same dose, and the effects exhibited an obvious dose-dependent manner.

As an initiator of excessive inflammation, TNF-*α*, which has a significant inflammatory effect, plays a major role in the inflammatory response. Similar to IL-1*β*, TNF-*α* can stimulate immune cells to produce more inflammatory factors and mediators through autocrine and paracrine mechanisms [28]. Previous investigations have demonstrated that the NF-*κ*B pathway plays the essential role in the inflammation-related diseases, and inhibition of the NF-*κ*B signaling transduction would be beneficial for the control and treatment of these inflammatory disorders [29, 30]. In the present study, the expression of NF-*κ*Bp65 and the content of TNF-*α* and IL-1*β* increased after LPS administration, suggesting that the expression of NF-*κ*Bp65 promoted the release of inflammatory mediators. BA-LP had an inhibitory effect on inflammatory factors in BALF, indicating that BA treats acute lung injury through anti-inflammatory effects.

Activation of the JNK-ERK signaling pathway is one of the early intracellular events after LPS acts on monocyte/macrophage, which is closely related to the synthesis and secretion of inflammatory cytokines [31]. It has been found that JNK-ERK signaling is involved in LPS-stimulated gene transcription and protein expression of inflammatory and anti-inflammatory cytokines. The JNK-ERK signaling pathway is necessary for the transcription and translation of TNF-*α* [32], and the activation of JNK is one of the conditions for TNF-*α* mRNA translation. ERK is the most important link of monocyte inflammatory response to LPS. The network inflammatory cascade produced by LPS is complex. The signal pathways endow the uniqueness of the biological reaction, which provides an alternative target for the treatment of LPS-induced inflammatory response. The present experiment showed that BA could reduce the key protein of the TLR4/JNK/ERK/NF-*κ*B signal pathway and block the transmission of the pathological signal.

In this paper, we found that the BA-LP had longer retention time and more concentration in lungs than BA in normal mice. In addition, pharmacological experiments showed that BA-LP had a better therapeutic effect on ALI than BA. BA can treat LPS-induced ALI through anti-inflammation by regulating the TLR4/JNK/ERK/NF-*κ*B signaling pathway. These findings suggest that BA-LP may be a new way for preventing and treating ALI. However, the occurrences and treatments of these diseases are complex. In the process of treatment of BA for ALI, other deep mechanisms may also be involved which needs further research.

## Figures and Tables

**Table 1:** Regression equations, correlation coefficients, and linear ranges of BA in mouse plasma and tissues.

Analyte	Regression equation	Correlation coefficients	Linear ranges (*μ*g/mL)
Plasma	*Y* = 0.224*X* + 0.05	1	4.8512-1516
Heart	*Y* = 0.208*X* + 0.048	1	1.4554-97.024
Liver	*Y* = 0.186*X* + 0.026	0.999	2.4256-194.048
Spleen	*Y* = 0.185*X* − 0.034	0.999	4.8512-194.048
Lung	*Y* = 0.174*X* + 0.027	0.999	2.4255-97.024
Kidney	*Y* = 0.164*X* − 0.135	0.999	2.4256-1516

**Table 2:** The results of intraday and interday precision and accuracy for BA in blank plasma and tissues (*n* = 5 for intra-day and *n* = 5^∗^3 for interday).

Analyte	Concentration (*μ*g/mL)	Intraday		Interday	
		Precision (%)	Accuracy (%)	Precision (%)	Accuracy (%)
Plasma	4.8512	5.80%	100.01%	5.46%	99.86%
	97.024	2.67%	99.37%	3.08%	99.00%
	1516	1.30%	99.06%	1.29%	98.46%

Heart	1.4554	4.26%	98.38%	5.01%	98.19%
	19.4048	2.44%	101.65%	3.40%	101.29%
	97.024	1.38%	99.90%	1.41%	100.02%

Liver	2.4256	3.15%	99.92%	4.58%	100.70%
	48.512	4.16%	98.25%	3.50%	98.46%
	194.048	1.29%	100.60%	1.20%	100.58%

Spleen	4.8512	2.99%	99.77%	6.13%	99.04%
	48.512	2.26%	99.64%	3.47%	99.85%
	194.048	1.04%	100.12%	1.10%	100.30%

Lung	2.4256	2.07%	100.97%	3.34%	100.04%
	19.40475	8.58%	95.32%	6.45%	98.97%
	97.024	2.45%	100.94%	7.51%	101.20%

Kidney	2.4256	4.73%	97.93%	4.58%	99.20%
	97.024	1.50%	99.73%	1.51%	99.86%
	1516	1.02%	98.67%	1.18%	98.63%

**Table 3:** Main pharmacokinetic parameters of plasma in normal mice after intravenous injection of BA-LP and BA.

Parameters	Units	BA-LP	BA
AUC_0-__*t*_	mg/L^∗^min	17536.084 ± 928.392	2712.911 ± 55.945
AUC_0-∞_	mg/L^∗^min	19574.508 ± 840.058	2958.151 ± 49.156
MRT_0-__*t*_	min	102.636 ± 1.749	17.354 ± 0.16
MRT_0-∞_	min	172.950 ± 14.110	41.167 ± 3.073
*t*_1/2_	min	202.963 ± 18.291	127.18 ± 10.849
*V*_d_	L/kg	1.500 ± 0.168	6.209 ± 0.582
CLz	L/min/kg	0.005	0.0338 ± 0.0004
*C*_max_	mg/L	192.689 ± 9.210	102.165 ± 1.788

BA-LP and BA were administered by intravenous injection at 100 mg/kg, and data were expressed as mean ± SD (*n* = 5).

**Table 4:** The concentrations of BA in tissues of normal mice after intravenous injection of BA-LP and BA.

Time (min)	Concentration in tissues (*μ*g/g)									
	Heart		Liver		Spleen		Lung		Kidney	
	BA-LP	BA	BA-LP	BA	BA-LP	BA	BA-LP	BA	BA-LP	BA
5	4.413 ± 0.592	2.977 ± 0.461	21.424 ± 1.190	17.025 ± 1.142	15.206 ± 1.098	3.685 ± 0.602	8.296 ± 1.332	5.493 ± 0.857	92.214 ± 9.867	226.611 ± 12.104
15	5.352 ± 1.047	3.871 ± 0.454	31.473 ± 1.481	18.570 ± 1.634	22.711 ± 2.024	9.012 ± 0.739	15.518 ± 2.138	7.590 ± 0.801	77.246 ± 6.525	53.969 ± 3.301
30	7.523 ± 0.732	2.499 ± 0.323	61.790 ± 1.411	5.041 ± 0.857	25.150 ± 1.384	7.494 ± 0.542	10.582 ± 1.167	3.877 ± 0.653	35.562 ± 3.484	14.073 ± 1.490
60	7.510 ± 0.599	0.444 ± 0.057	50.343 ± 1.814	1.931 ± 0.074	49.719 ± 2.111	5.014 ± 0.251	15.530 ± 0.924	2.096 ± 0.540	17.945 ± 2.196	4.636 ± 0.282
90	5.544 ± 0.551	0.385 ± 0.048	18.440 ± 1.187	1.003 ± 0.042	16.511 ± 1.561	4.728 ± 0.198	8.417 ± 1.110	1.326 ± 0.041	9.761 ± 0.747	3.560 ± 0.200
120	1.733 ± 0.248	ND	2.245 ± 0.181	0.451 ± 0.037	8.087 ± 0.691	ND	3.690 ± 0.823	0.702 ± 0.037	5.483 ± 0.335	2.416 ± 0.181
240	1.279 ± 0.098	ND	4.265 ± 0.466	ND	6.552 ± 0.423	ND	3.598 ± 0.422	ND	4.432 ± 0.289	ND
360	1.073 ± 0.143	ND	3.127 ± 0.502	ND	5.894 ± 0.572	ND	1.772 ± 0.126	ND	3.766 ± 0.303	ND
480	0.868 ± 0.083	ND	1.355 ± 0.057	ND	5.251 ± 0.654	ND	1.557 ± 0.099	ND	3.045 ± 0.151	ND

BA-LP and BA were administered by intravenous injection at 100 mg/kg, and data were expressed as mean ± SD (*n* = 5).

**Table 5:** The targeting parameters in tissues of normal mice after intravenous injection of BA-LP and BA.

Tissue	Te_(BA-LP)_ (%)	Te_(BA)_ (%)	TI	Ce	RTE (%)
Heart	86.168	34.953	31.260	3.327	1.465
Liver	3.854	2.684	9.508	2.065	0.436
Spleen	36.228	15.606	13.196	5.869	1.321
Lung	7.266	6.531	7.401	2.123	0.113
Kidney	19.956	210.919	1.643	0.407	-0.905

BA-LP and BA were administered by intravenous injection at 100 mg/kg, and data were expressed as mean ± SD (*n* = 5).

## Data Availability

The data used to support the findings of this study are included within the article.

## Additional Points

*Highlights*. The retention time of baicalin liposome is longer than that of the baicalin monomer, and the concentration in the lung is higher in vivo. Baicalin liposome can protect against lipopolysaccharide-induced acute lung injury in mice by an anti-inflammatory effect at a dosage-dependent manner. The expression of key proteins in the TLR4/NF-*κ*B and JNK/ERK signaling pathways were upregulated after the treatment of baicalin.

## Supplementary Materials

Supplementary Materials Graphical abstract: Tissues distributions of BA-LP in normal mice and the intervention of BA-LP on LPS- induced ACL in mice.
