# Supplementary material for: Baicalin Liposome Alleviates Lipopolysaccharide-Induced Acute Lung Injury in Mice via Inhibiting TLR4/JNK/ERK/NF-κB Pathway
Source: Mediators Inflamm. 2020 Nov 11;2020:8414062. doi: 10.1155/2020/8414062 (PMC7673921; doi:10.1155/2020/8414062)
Supplement: Supplementary Materials — Graphical abstract: Tissues distributions of BA-LP in normal mice and the intervention of BA-LP on LPS- induced ACL in mice. [file 8414062.f1.docx]

Dear editor:

Thank you for your advice. The supplementary material I submitted is my graphic abstract. This picture mainly explains the main content of my experiment, so I also upload it together. Thank you.
